# Associations between religious/spiritual beliefs and behaviours and dietary patterns: analysis of the parental generation in a prospective cohort study (ALSPAC) in Southwest England

**DOI:** 10.1017/S1368980023001866

**Published:** 2023-12

**Authors:** Daniel Major-Smith, Jimmy Morgan, Pauline Emmett, Jean Golding, Kate Northstone

**Affiliations:** 1 Centre for Academic Child Health, Population Health Sciences, Bristol Medical School, University of Bristol, Bristol, BS8 2BN, UK; 2 MRC Integrative Epidemiology Unit, University of Bristol, Bristol, UK; 3 Population Health Sciences, Bristol Medical School, University of Bristol, Bristol, UK

**Keywords:** ALSPAC, Religion, Diet, Dietary patterns, Nutrient intake

## Abstract

**Objective:**

Religious/spiritual beliefs and behaviours (RSBB) have been associated with health outcomes, with diet a potential mediator of this relationship. We therefore explored whether RSBB were associated with differences in diet.

**Design:**

Dietary patterns and nutrient intakes were derived from food frequency questionnaire completed by pregnant women in 1991–1992 (mean age = 28·3 years, range = 15–46) and by the mothers and partners 4 years post-partum (mothers mean age = 32·3, range = 19–49; partners mean age = 34·5, range = 18–74). RSBB exposures measured in pregnancy included religious belief, affiliation and attendance. We first explored whether RSBBs were associated with dietary patterns in confounder-adjusted linear regression models. If associations were found, we examined whether RSBB were associated with nutrient intake (linear regression) and following nutrient intake guidelines (logistic regression).

**Setting:**

Prospective birth cohort study in Southwest England (Avon Longitudinal Study of Parents and Children; ALSPAC).

**Participants:**

13 689 enrolled mothers and their associated partners.

**Results:**

In pregnant women, RSBB were associated with higher ‘traditional’ (i.e. ‘meat and two veg’) and lower ‘vegetarian’ dietary pattern scores. Religious attendance and non-Christian religious affiliation were associated with higher ‘health-conscious’ dietary pattern scores. Religious attendance was associated with increased micronutrient intake and following recommended micronutrient intake guidelines, with weaker effects for religious belief and affiliation. Comparable patterns were observed for mothers and partners 4 years post-partum, although associations between RSBB and nutrient intakes were weaker for partners.

**Conclusions:**

RSBBs are associated with broad dietary patterns and nutrient intake in this cohort. If these reflect causal relationships, diet may potentially mediate the pathway between RSBB and health.

Numerous observational studies have suggested that religious/spiritual beliefs and behaviours (RSBB), such as belief in God and attendance at a place of worship, are associated with both physical and mental health, such as lower rates of suicide, depression and overall mortality^(1–3)^. However, due to the impossibility of conducting randomised controlled trials these studies are by necessity observational, meaning that causal relationships are difficult to establish with certainty; nonetheless, longitudinal studies with repeated measurements and data on relevant confounders do suggest that some of these associations may be causal^(4)^.

Assuming these associations are causal, a key next step is to understand the mediators of these relationships. Currently, these are not fully known but likely include factors such as social support and norms encouraging health-promoting behaviours (e.g. reductions in smoking/alcohol/drug use^(1)^). One further possible mediator on the causal pathway between RSBB and health is diet. Diet can impact health in several ways, such as diets high in fat and sugar and low in fruits/vegetables altering the risk of obesity, hypertension, type 2 diabetes, CVD, various cancers (especially breast) and mortality^(5,6)^. Diet in pregnancy, and fish intake in particular, has also been associated with child outcomes, such as preterm birth, birth weight and child neurocognitive development^(7–9)^.

Many societies have norms and taboos surrounding food^(10)^, which are often linked to group and individual identity^(11)^ and in some cases may serve adaptive functions such as avoiding toxins during pregnancy^(12)^ or as a signal of one’s commitment to the group^(13)^. Religions also frequently have restrictions regarding diet, such as avoiding pork in Judaism and Islam, or advocating vegetarian diets in Hinduism and Buddhism^(2,14)^. Additionally, as religions frequently promote healthy behaviours, such as avoiding smoking, alcohol or drugs^(1,2)^, these health-promoting norms may also translate into a healthier diet. We may therefore predict a positive association between RSBB and diet and potentially downstream health effects.

As the main religion in the study population in this article (UK) is Christianity, we will focus on associations between Christianity and diet here. Compared with other religions, Christianity appears to have fewer dietary restrictions^(14)^. One exception is Lent (a period of fasting for 40 days prior to Easter) which has traditionally been associated with dietary restrictions and avoidance of certain foods (such as meat and other animal products), but as the UK and other Western societies have become more secular the observance of both fasting and avoidance of certain animal products has declined. Additionally, as Lent only lasts for just over a month, long-term health impacts may be minimal. Catholicism and other Christian denominations – including Anglican/Church of England and Methodist – have also traditionally had a ‘Friday fast’ and avoided animal products and alcohol on Fridays in remembrance of Jesus’ crucifixion, but where eating fish on Fridays is permitted. If certain Christian denominations do encourage the eating of fish, this may boost omega-3 (*n*-3) fatty acid intake and many other important minerals (e.g. iodine) and vitamins (e.g. A and D), which have been associated with various health outcomes. For instance, eating fish in pregnancy has been associated with enhanced pregnancy outcomes (lower risk of preterm birth and a small birth weight increase^(15)^), and child neurocognitive development^(8,16)^, but may increase the risk of rapid child growth and obesity^(17)^. However, the extent to which these religious practices translate into observable dietary differences is unclear; in general, Christians (in the USA, based on a small self-selected sample) viewed behaviours such as drug use, smoking and alcohol consumption as more ‘sinful’ than other health-related behaviours such as poor diet, physical inactivity and obesity^(18)^, suggesting that Christian proscriptions surrounding diet may be relatively weak.

Research on the associations between religion and diet has so far been mixed, with the majority of work focusing on Christianity. Some studies report an association between RSBB and a putatively healthier diet^(19–21)^, others null effects^(22)^, while others find mixed or inconsistent results, such as findings specific to one sex^(23)^. A summary of studies published up until 2012^(2)^ reported that, of twenty studies looking at RSBB and diet, 12 (60 %) found that RSBB was associated with a healthier diet or greater nutritional intake, while others had mixed or null findings (plus one where results were in the opposite direction). The authors conclude that there may be preliminary evidence for an association between diet and religion, but further, higher-quality, studies are necessary. For example, many of these previous studies used crude and self-reported diet variables, cross-sectional rather than longitudinal data collection, small sample sizes, a focus on Christian participants and with most studies conducted in the USA.

There is suggestive theoretical and empirical evidence that religion may shape diet, with potential downstream effects on health and mortality, although the current evidence is somewhat mixed and based on studies with several limitations. We therefore aimed to explore this topic in detail using secondary data from the Avon Longitudinal Study of Parents and Children (ALSPAC), a large prospective birth cohort study centred in Bristol, UK. Our research question aimed to assess whether RSBB is associated with, and may potentially cause, differences in diet. This adds to the evidence base of these previous studies, while extending it in several ways, including: prospective data collection (avoiding recall bias), large sample size (*n* 13 689), geographically representative and with detailed and repeatedly collected diet data. Specifically, we explored whether various facets of RSBB (belief in God/divine power, religious affiliation and religious service attendance) were associated with dietary patterns and nutrient intakes in ALSPAC parents (mothers and partners). These analyses were undertaken on women, both during pregnancy and approximately 4 years post-partum, and on their partners 4 years post-partum.

## Methods

### Participants

Pregnant women resident in Avon, UK with expected dates of delivery between 1st April 1991 and 31st December 1992 were invited to participate in the study. The initial number of pregnancies enrolled was 14 541, of which there were a total of 14 676 fetuses, resulting in 14 062 live births and 13 988 children alive at 1 year of age; for more information on ALSPAC recruitment, see the associated cohort profile papers^(24,25)^. The current research focuses specifically on the parents of the study child. After removing pregnancies that did not result in a live birth (most being early miscarriages; 674 observations), removing one pregnancy if the mother had two pregnancies enrolled in ALSPAC (157 observations) and dropping observations for participants who had withdrawn consent for their data to be used (twenty-one observations), a total of 13 689 mothers were included in the final dataset.

For each mother, we also included their associated partner, usually the father of the study child. Partners/fathers (hereafter ‘partners’) were not formally enrolled into ALSPAC but were given partner-based questionnaires by the mother (if she had a partner and chose to invite them)^(26)^. This means that partner-based questionnaires may not have been completed by the same partner over time (although numbers of such cases are likely to be relatively small); for the purposes of this study, we assume that the identity of the partner is the same over all waves of data collection used. Note also that although many partners never participated in ALSPAC (approx. 2000), all potential partners have been included here as we have information about many of them based on questionnaires completed by the mother about the partner.

The study website contains details of all the data that are available through a fully searchable data dictionary and variable search tool:

http://www.bristol.ac.uk/alspac/researchers/our-data/.

### Outcome measures: dietary patterns and nutrient intakes

Dietary data were derived from food frequency questionnaires (FFQ), asked via questionnaires during pregnancy (approximately 32 weeks gestation; mothers only (mean age = 28·3, range = 15–46)) and approximately 4 years post-partum (mothers (mean age = 32·3, range = 19–49) and partners (mean age = 34·5, range = 18–74); although partners completed a FFQ in pregnancy, not all FFQ questions were asked, hence its exclusion here). For an overview of the ALSPAC diet data collections, see^(27)^; here, we only provide a brief description of these measures.

For each FFQ, participants answered a series of questions regarding the frequency of food and drink consumption (never or rarely *v* once in 2 weeks *v* 1–3 times a week *v* 4–7 times a week *v* more than once a day). For some foods, usually consumed daily, the type was also recorded (e.g. wholemeal *v* brown/granary *v* white bread and full fat *v* semi-skimmed *v* skimmed milk). Using standard portion sizes and UK food nutrient content databases, this FFQ was used to calculate approximate daily intake for a range of nutrients^(27,28)^. FFQ data were also used to derive underlying dietary patterns using principal components analysis^(29)^ during pregnancy^(30)^, and in the mothers^(31)^ and partners^(32)^ 4 years post-partum. These dietary pattern scores were used to simplify the data from a large number of individual food items to a smaller, more manageable, number of dimensions which reflect overall patterns of dietary intake, an approach which is increasingly common in nutritional epidemiology^(29)^; for more details on these methods, and their validity and reliability, see^(29–33)^. Dietary pattern scores are approximately normally distributed and standardised with a mean of 0 and SD of 1. A summary of these dietary principal components is provided in Table 1 (note that as these were data-driven, they vary by time/person-point).

**Table 1:** Description of dietary patterns used in the present study

Dietary pattern	Description and example foods	Mothers in pregnancy (32 weeks gestation)	Mothers 4 years post-partum	Partners 4 years post-partum
‘Health-conscious’	*‘Healthy’ diet* Non-white bread, wholegrain breakfast cereal, fish, pulses, pasta, rice, salad, fruit	Yes	Yes	Yes
‘Traditional’	*‘Meat and 2 veg’ diet* Vegetables, potatoes, red meat and poultry	Yes	No	Yes
‘Processed’	*High-fat processed food diet* Meat pies, sausages, burgers, fried foods, pizza, chips and baked beans	Yes	Yes	No
‘Confectionery’	*High-sugar intake diet* Chocolate, sweets, biscuits, cakes and other puddings	Yes	Yes	No
‘Processed/Confectionery’	*Combination of high-fat processed and high-sugar diet* Chocolate, sweets, biscuits, cakes, other puddings, meat pies, sausages, pizza, chips and baked beans	No	No	Yes
‘Vegetarian’	*‘Vegetarian’ diet* Meat substitutes, pulses, nuts (negative loadings for red meat and poultry)	Yes	Yes	No
‘Semi-vegetarian’	*Similar to vegetarian, but fewer negative loadings for meat* Vegetarian pies, meat substitutes, pulses, nuts, pizza, fish	No	No	Yes

Note that dietary patterns differ somewhat by time/person-point, and even if the same broad dietary principal component was identified the example foods and weightings may differ slightly between the time points (see^(29)^).

Both the dietary pattern scores and the estimated nutrient intake data from each of the three time/person-points were our continuous outcome variables. In addition, we recoded the continuous nutrient measures into binary variables indicating whether participants met UK government recommended nutrient intake guidelines (using information from the Committee on Medical Aspects of Food Policy report (COMA^(34)^) or Scientific Advisory Committee on Nutrition 2016 recommendations (SACN; https://assets.publishing.service.gov.uk/government/uploads/system/uploads/attachment_data/file/618167/government_dietary_recommendations.pdf)). Details of the nutrients and male and female guidelines are in see online supplementary material, Supplemental Table 1.

### Exposures measures: religious/spiritual beliefs and behaviours

The majority of mother and partner RSBB variables were collected during pregnancy, with the remainder (approx. 10 %) collected 4 months after birth, via questionnaires. Given the complexities and multi-faceted nature of religious belief^(2,35)^, we explore three broad self-reported exposure variables: (1) belief in God/divine power (yes *v* not sure *v* no); (2) religious affiliation (Christian *v* none *v* other faith/belief) and (3) frequency of attendance at a place of worship (at least once a month *v* at least once a year *v* not at all). These RSBB variables cover a range of theoretically relevant religious beliefs and behaviours^(36)^, have been used extensively in previous research, both in ALSPAC^(37,38)^ and more widely^(39)^, and have been described in detail previously^(40)^.

### Confounder variables

The following variables hypothesised to cause both the exposure (RSBB) and the outcome (dietary patterns and nutrient intakes) were chosen as confounders: age at completion of FFQ, parity, urban/rural location, marital status, ethnicity and multiple proxies for socio-economic position (highest education attainment, occupational social class, area-level index of multiple deprivation, housing status and recent financial problems; see online supplementary material, Supplemental Table 2 for full details of these variables). Other than ‘age at completion’ 4 years post-partum, all confounders were measured in pregnancy.

Our choice of confounders was predominantly based on existing literature^(2,3,30,32,41)^, which have identified these factors as potential causes of both RSBB and dietary patterns. Our hypothesised causal structure of the data is represented in online supplementary material, Supplemental Fig. 1. We did not include other potential causes of dietary patterns, such as smoking, alcohol intake or mental health status, as confounders in these analyses; current research suggests that these are more likely to be causes of RSBB^(3,4)^, and hence mediators on the causal pathway from RSBB to dietary patterns, and their inclusion may bias the total effect estimate between RSBB and dietary patterns^(42,43)^. However, given that the true causal relationships between these variables are largely unknown – we are assuming that marital status causes RSBB, rather than vice versa, for instance – we note that these causal assumptions may not reflect the true confounding structure of the data but rather are our best efforts to appropriately address bias due to potential confounding.

In addition to the confounders detailed above, for the nutrient intake analyses we also performed sensitivity analyses including ‘total energy intake’ as a covariate in the adjusted models to control for differences in energy intake^(44)^. These sensitivity analyses were performed because it is not clear whether potential differences in energy intake are mediators on the causal pathway between RSBB and nutrient intake – for example, religious individuals may have different metabolic demands/total food consumption and hence have differential nutrient intake (in which case total energy intake should not be adjusted for; this is our main analysis) – or whether energy intake is a confounder – for example, religious individuals may under- or over-report consumption, or other confounding factors may cause both RSBB and energy intake (in which case total energy intake should be adjusted for; this is our sensitivity analysis). By comparing results with *v* without this total energy intake covariate, we can explore if and how results differ and assess how robust these results are to different model specifications and assumptions. Note that analyses of dietary patterns did not include this covariate, as adjustment for total energy intake is not required^(45)^.

### Analysis

We explored the associations between RSBB and the dietary pattern scores in complete-case unadjusted, complete-case adjusted and multiple-imputed adjusted models (see below for details on missing data and multiple imputation). A Bonferroni correction corresponding to number of outcome comparisons was used in an attempt to avoid false positive outcomes within each block of tests (e.g. in the pregnancy data there were five dietary patterns, giving a threshold when using a standard 0·05 *α* value of 0·05/5 = 0·01). However, rather than using these thresholds to arbitrarily dichotomise results into ‘significant’ and ‘non-significant’^(46)^, we use these *P*-values to assess the strength of evidence against the null hypothesis of no difference by RSBB^(47)^.

If associations between RSBB and dietary patterns were identified within a time point/participant group, then nutrient intakes and recommended nutrient intake thresholds were examined to explore these dietary differences in more detail (previous research has demonstrated that these dietary principal components in pregnancy are associated with nutrient intake^(33)^). As above, these analyses were repeated using complete-case unadjusted, complete-case adjusted and multiple-imputation adjusted models, with appropriate Bonferroni correction for multiple comparisons. For reasons of space and clarity, full details of these ‘nutrient intake’ results will predominantly be given in the supplementary information. All analyses were conducted in Stata v.17.

### Missing data

Missing data may result in biased associations if the outcome, or the outcome and the exposure, are related to missingness^(42,48,49)^. Individual variables collected early in ALSPAC have relatively little missing data (usually <20 % for mothers in pregnancy); however, attrition over time combined with data originating from different questionnaires (which not all participants completed) can lead to substantial levels of missing data in complete-case analyses which may result in bias.

As a robustness check for our complete-case analysis results, we performed multiple imputation via chained equations to impute missing data^(49–51)^. In addition to all covariates in the substantive analysis model, we also included additional auxiliary variables in the imputation model, which predict the outcomes, exposures and missingness in these variables. These auxiliary variables included RSBB and dietary pattern data from other sources (e.g. using mother’s dietary patterns in pregnancy to impute patterns 4 years post-partum), additional proxies for socio-economic position and other factors associated with RSBB, dietary patterns and missing data (discussed in more detail below). We assumed that the inclusion of these auxiliary variables met the ‘missing at random’ assumption required for unbiased imputation estimates, although we cannot rule out residual bias due to data being ‘missing not at random’.

For the dietary pattern data, we imputed up to the total cohort (*n* 13 689) for both mothers and partners. Given that the patterns of missing data were largely arbitrary (i.e. small amounts of missing data on many variables), multiple imputation is likely to be effective as it can use much of the observed data from other variables to impute missing values. Even if complete-case analyses are unbiased, making use of this additional information is likely to improve efficiency (i.e. estimating the parameters of interest more accurately). For instance, in the mother’s pregnancy data, of 11 843 participants with dietary pattern data, only 7780 complete cases remained in the adjusted model where ‘belief in God/divine power’ was the exposure, a loss of approximately one-third of the data.

For the nutrient data, due to the large number of nutrient variables^(33)^, we decided to impute missing data in the RSBB exposures and confounders but not the nutrient outcomes (*n* 11 812 for mothers in pregnancy; *n* 9296 for mothers 4 years post-partum; *n* 4786 for partners 4 years post-partum); this is because imputing thirty-three nutrient outcomes, in addition to all exposures, confounders and auxiliary variables, would have been prohibitive in terms of computer processing time.

For all imputation models, we performed fifty imputations with a burn-in period of ten iterations. Convergence plots were examined to ensure that a steady state had been reached and that chains were well-mixed; if chains were not well-mixed, the burn-in period was increased to twenty iterations. Imputations were performed using appropriate models (e.g. linear, logistic, ordinal, multinomial), with continuous variables determined to deviate from a normal distribution imputed using predictive mean matching using the five nearest neighbours^(51)^. To avoid perfect prediction of categorical variables during imputation, we used the ‘augment’ command^(52)^. Analyses on these imputed datasets were combined using Rubin’s Rules. Additional discussion of the imputation models is provided in section S1 of the supplementary information.

## Results

### Descriptive statistics and missing data

Descriptive statistics for the RSBB exposures are in Table 2. Regarding religious affiliation, of mothers categorised as Christian 80 % were Protestant/Church of England, 10 % Roman Catholic and 10 % other denominations, while mothers with ‘other’ religious affiliations predominantly self-identified as ‘other’ (73 %), with the remaining mothers including small numbers of Jewish, Buddhist, Sikh, Hindu, Muslim and Rastafarian participants. Similar proportions were reported for partners.

**Table 2:** Descriptive statistics for the religious/spiritual beliefs and behaviour variables measured in pregnancy (*n* 13 689)

		Mother (*n*)	%	Partner (*n*)	%
Belief in God/divine power	Yes	6029	49·9	3521	36·9
	Not sure	4262	35·3	3296	34·5
	No	1791	14·8	2737	28·6
	Total	12 082		9554	
Missing data		1607	11·7	4135	30·2
Religious affiliation	None	1824	15·3	2425	25·8
	Christian	9604	80·5	6480	68·9
	Other	508	4·3	498	5·3
	Total	11 936		9403	
Missing data		1753	12·8	4286	31·3
Attendance at church/place of worship	Min once a month	1684	14·3	967	10·3
	Min once a year	3461	29·3	2450	26·2
	Not at all	6667	56·4	5940	63·5
	Total	11 812		9357	
Missing data		1877	13·7	4332	31·7

In total, 11 843 mothers have dietary pattern data in pregnancy (13·5 % missing), 9379 mothers have dietary pattern data 4 years post-partum (31·5 % missing) and 4630 partners have dietary pattern data 4 years post-partum (66·2 % missing). Descriptive statistics for the nutrient intakes and recommended daily nutrient intake binary variables for each time point are in online supplementary material, Supplemental Tables 3 to 5; as they were based on the same FFQ data, sample sizes and proportions of missing data were similar – although not exactly identical – for both dietary pattern scores and nutrient intakes.

Approximately 35 % of mothers and 44 % of partners had either A-level (optional qualifications undertaken at age 18) or degree-level qualifications. Ninety-seven percent of mothers and 96 % of partners were of a White ethnic background, and the majority of participants owned their own home (73 %) and were currently married (75 %). For full details of study characteristics, confounders and auxiliary variables used for multiple imputation, see online supplementary material, Supplemental Table 2.

### Mother’s diet in pregnancy

#### Dietary patterns

**Fig. 1:**
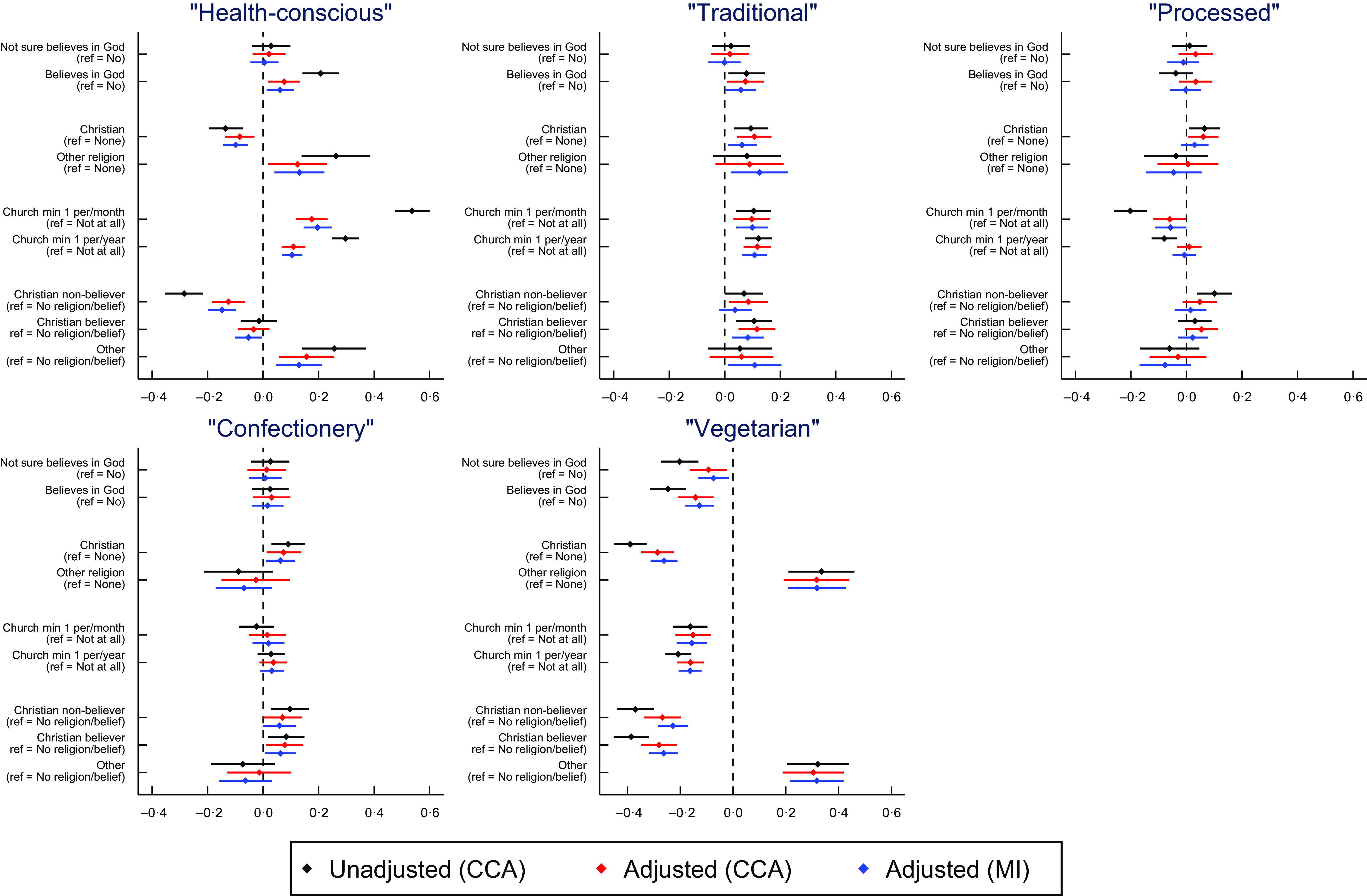
Associations between religious/spiritual beliefs and behaviours and dietary patterns from mothers in pregnancy. Positive coefficients indicate a higher score. Error bars denote 95 % CI. CCA = complete-case analysis; MI = multiple imputation. *n* for belief in God/divine power CCA = 7780; *n* for religious affiliation CCA = 7695; *n* for attendance at a church/place of worship = 7620; *n* for religious belief and affiliation combined = 7677; *n* for multiple imputation = 13 689

Results for the dietary patterns components are displayed in Fig. 1. There is little difference in the parameter estimates between the adjusted complete-case models using just the observed data and the imputed data (although the standard errors of the latter are slightly smaller, indicating increased efficiency), so here we will focus on the imputed results. Full results are in online supplementary material, Supplemental Table 6.

Taking the ‘health-conscious’ component first, belief in God/divine power was somewhat associated with higher ‘health-conscious’ dietary pattern scores relative to those who do not believe (*β* = 0·062, 95 % CI (0·013, 0·110), *P* = 0·013). However, Christian affiliation was associated with lower ‘health-conscious’ pattern scores compared with those with no religious affiliation (*β* = -0·099, 95 % CI (−0·144, −0·054), *P* < 0·001). To examine this seemingly contradictory pattern of results in more detail, we explored belief in God/divine power and Christian affiliation together: identifying as Christian but no/not sure regarding belief in God was associated with lower ‘health-conscious’ scores relative to non-religious non-believers (*β* = -0·148, 95 % CI (−0·198, −0·098), *P* < 0·001), while Christians who believed in God only had marginally lower ‘health-conscious’ scores (*β* = -0·053, 95 % CI (−0·101, −0·005), *P* = 0·030). Relative to not attending, more frequent attendance at a place of worship was associated with higher ‘health-conscious’ scores (minimum once a year: *β* = 0·104, 95 % CI (0·067, 0·141), *P* < 0·001; minimum once a month: *β* = 0·196, 95 % CI (0·146, 0·247), *P* < 0·001). Other strong effects of RSBB were found for the ‘vegetarian’ component, with belief in God/a divine power (yes: *β* = −0·127, 95 % CI (−0·182, −0·072), *P* < 0·001; not sure: *β* = −0·074, 95 % CI (−0·131, −0·016), *P* = 0·012), Christian affiliation (*β* = −0·261, 95 % CI (0·312, −0·210), *P* < 0·001) and religious attendance (minimum once a year: *β* = -0·162, 95 % CI (−0·206, −0·119), *P* < 0·001; minimum once a month: *β* = -0·156, 95 % CI (−0·214, −0·099), *P* < 0·001) all associated with lower ‘vegetarian’ dietary pattern scores. Having a non-Christian religious affiliation was associated with higher ‘vegetarian’ (*β* = 0·318, 95 % CI (0·207, 0·428), *P* < 0·001) and ‘health conscious’ (*β* = 0·131, 95 % CI (0·041, 0·221), *P* = 0·004) dietary pattern scores. Associations with RSBB were weaker for the ‘traditional’ component, although RSBB was somewhat associated with higher ‘traditional’ dietary pattern scores (religious belief (yes): *β* = 0·058, 95 % CI (0·002, 0·113), *P* = 0·042; Christian affiliation: (*β* = 0·063, 95 % CI (0·011, 0·115), *P* = 0·018); attend minimum once a year: (*β* = 0·107, 95 % CI (0·064, 0·151), *P* < 0·001); attend minimum once a month: (*β* = 0·099, 95 % CI (0·042, 0·156), *P* = 0·001)); few associations were found for ‘processed’ or ‘confectionery’ components.

#### Nutrient intakes

As RSBB was found to be associated with dietary pattern scores for mothers during pregnancy, we explored whether RSBB was associated with specific nutrient intakes (see online supplementary material, Supplemental Figs 2 and 3; Table 3). Detailed results are provided in section S2 of the supplementary information, but in summary we observed that RSBB, and religious attendance in particular, were positively associated with micronutrient intakes (full results in online supplementary material, Supplemental Tables 7–10). Religious attendance – although less so for other RSBB exposures – was also associated with being more likely to follow recommended nutritional intake guidelines regarding micronutrients, although fewer associations were reported compared with overall nutrient intake (full results in online supplementary material, Supplemental Tables 11–14). Associations with nutrient intakes and following nutrient intake guidelines were similar when including ‘total energy intake’ as a covariate, although some associations were attenuated towards the null, particularly for religious attendance (online supplementary material, Supplemental Table 15, Figs 4 and 5; full results in online supplementary material, Supplemental Tables 16–23). For all time/person-points, a summary of the associations between RSBB and nutrient intake can be found in Table 3, with breakdowns on a nutrient-by-nutrient basis in Tables 4 (for overall nutrient intake) and 5 (for following nutrient intake guidelines; see online supplementary material, Supplemental Tables 15, 24 and 25, respectively, for equivalent summaries when adjusting for total energy intake).

**Table 3:** Summary of associations between RSBB and nutrient intake for mothers in pregnancy and mothers and partners 4 years post-partum

		Mothers in pregnancy								Mothers at 4 years post-partum								Partners at 4 years post-partum							
		Nutrient intake (*n* 33)				Follow recommended daily intake (*n* 29)				Nutrient intake (*n* 33)				Follow recommended daily intake (*n* 28)				Nutrient intake (*n* 33)				Follow recommended daily intake (*n* 24)			
		Bon-adj (0·0015)		0·05		Bon-adj (0·0017)		0·05		Bon-adj (0·0015)		0·05		Bon-adj (0·0018)		0·05		Bon-adj (0·0015)		0·05		Bon-adj (0·0021)		0·05	
		*n*	%	*n*	%	*n*	%	*n*	%	*n*	%	*n*	%	*n*	%	*n*	%	*n*	%	*n*	%	*n*	%	*n*	%
Belief in God (ref = no)	Not sure	0	0	0	0	0	0	4	14	0	0	3	9	0	0	4	14	0	0	1	3	0	0	0	0
	Yes	7	21	19	58	2	7	11	38	13	39	29	88	6	21	14	50	0	0	4	12	0	0	3	13
Religious affiliation (ref = none)	Christian	1	3	10	30	1	3	1	3	6	18	13	39	0	0	3	11	0	0	0	0	0	0	0	0
	Other	0	0	5	15	0	0	2	7	2	6	12	36	0	0	1	4	0	0	4	12	0	0	0	0
Belief and religion (ref = none)	Christian believer	4	12	13	39	0	0	4	14	13	39	20	61	2	7	10	36	0	0	1	3	0	0	0	0
	Christian non-believer	4	12	6	18	2	7	4	14	0	0	6	18	0	0	3	11	0	0	0	0	0	0	0	0
	Other	0	0	8	24	0	0	3	11	7	21	14	42	1	4	4	14	0	0	3	9	0	0	1	4
Attendance at place of worship (ref = not at all)	Min once a month OR once a year	30	91	32	97	18	62	24	83	29	88	33	100	14	50	24	86	8	24	26	79	1	4	9	38

RSBB, religious/spiritual beliefs and behaviours.

Results are from adjusted analyses on the imputed data up to number with nutrient data; *n* for mother in pregnancy = 11 812; *n* for mother 4 years post-partum = 9296; *n* for partner 4 years post-partum = 4786. The table displays the number of associations meeting both the Bonferroni-corrected *α* threshold and convention 0·05 *α* levels, although we stress that these cut-offs are arbitrary and are intended only to provide a broad summary of the data. Note that for attendance at a place of worship, results for minimum once a month and minimum once a year have been combined together. Bon-adj = Bonferroni-corrected *α* value (the adjusted *α* level is given in brackets).

**Table 4:** Summary of results for overall intake of thirty-three nutrients

	Belief in God (ref = no)						Religious affiliation (ref = none)						Belief and religious affiliation (ref = non-religious non-believer)									Attendance at place of worship (ref = not at all)		
	Not sure			Yes			Christian			Other			Christian non-believer			Christian believer			Other			Min once a month/min once a year		
	Preg	M4	P4	Preg	M4	P4	Preg	M4	P4	Preg	M4	P4	Preg	M4	P4	Preg	M4	P4	Preg	M4	P4	Preg	M4	P4
Energy (kJ)	–	–	–	–	*Y+*	–	–	–	–	–	–	–	–	–	–	–	*Y+*	–	–	*Y+*	–	**Y+**	**Y+**	*Y+*
Carbohydrate (g)	–	–	–	–	*Y+*	–	–	–	–	–	*Y+*	–	–	–	–	–	**Y+**	–	–	*Y+*	–	**Y+**	**Y+**	*Y+*
Total sugars (g)	–	–	–	–	*Y+*	–	–	**Y+**	–	–	–	–	–	–	–	–	**Y+**	–	–	–	–	**Y+**	**Y+**	–
Free sugars (g)	–	–	–	–	*Y+*	–	–	**Y+**	–	–	–	–	–	*Y+*	–	–	**Y+**	–	–	–	–	–	*Y+*	–
Starch (g)	–	–	–	–	*Y+*	–	–	–	–	–	*Y+*	–	–	–	–	–	–	–	–	*Y+*	–	**Y+**	**Y+**	*Y+*
NSP (fibre) (g)	–	–	*Y+*	–	*Y+*	*Y+*	*Y*-	–	–	*Y+*	*Y+*	*Y+*	**Y**-	*Y*-	–	–	–	–	*Y+*	**Y+**	*Y+*	**Y+**	**Y+**	**Y+**
Fat (g)	–	–	–	–	*Y+*	–	–	–	–	–	–	–	–	–	–	–	–	–	–	–	–	**Y+**	**Y+**	*Y+*
Monounsaturated fat (g)	–	–	–	–	*Y+*	–	–	–	–	–	–	–	–	–	–	–	*Y+*	–	–	–	–	**Y+**	**Y+**	*Y+*
Polyunsaturated fat (g)	–	–	–	*Y+*	–	–	–	–	–	–	*Y+*	–	–	*Y*-	–	–	–	–	–	*Y+*	–	**Y+**	*Y+*	–
Saturated fat (g)	–	–	–	–	–	–	–	–	–	–	–	–	–	–	–	–	–	–	–	–	–	*Y+*	**Y+**	*Y+*
*n*-3 fatty acid from fish (g)	–	–	–	*Y+*	*Y+*	–	–	–	–	–	–	*Y+*	–	–	–	–	*Y+*	–	–	–	–	**Y+**	**Y+**	–
Cholesterol (mg)	–	–	–	–	**Y+**	–	*Y+*	*Y+*	–	–	–	–	–	–	–	*Y+*	**Y+**	–	–	–	–	**Y+**	**Y+**	*Y+*
Protein (g)	–	*Y+*	–	**Y+**	**Y+**	–	*Y+*	**Y+**	–	–	–	–	–	–	–	**Y+**	**Y+**	–	–	–	–	**Y+**	**Y+**	*Y+*
Thiamin (mg)	–	–	–	**Y+**	–	–	–	–	–	–	*Y+*	*Y+*	–	–	–	*Y+*	–	–	*Y+*	**Y+**	*Y+*	**Y+**	**Y+**	**Y+**
Riboflavin (mg)	–	–	–	*Y+*	**Y+**	–	*Y+*	*Y+*	–	–	–	–	–	–	–	**Y+**	**Y+**	–	–	*Y+*	–	**Y+**	**Y+**	*Y+*
Niacin eq. (mg)	–	*Y+*	–	**Y+**	**Y+**	–	*Y+*	**Y+**	–	–	–	–	–	–	–	**Y+**	**Y+**	–	–	–	–	**Y+**	**Y+**	*Y+*
Vitamin B_6_ (mg)	–	–	–	**Y+**	**Y+**	–	**Y+**	**Y+**	–	–	–	–	–	–	–	**Y+**	**Y+**	–	–	–	–	**Y+**	**Y+**	**Y+**
Vitamin B_12_ (µg)	–	*Y+*	–	*Y+*	**Y+**	–	*Y+*	**Y+**	–	–	–	–	–	–	–	*Y+*	**Y+**	–	–	–	–	**Y+**	**Y+**	**Y+**
Folate (µg)	–	–	–	*Y+*	**Y+**	–	–	–	–	*Y+*	*Y+*	–	–	–	–	*Y+*	*Y+*	–	*Y+*	**Y+**	–	**Y+**	**Y+**	*Y+*
Vitamin C (mg)	–	–	–	–	*Y+*	*Y+*	–	*Y+*	–	*Y+*	**Y+**	–	–	–	–	–	*Y+*	*Y+*	*Y+*	**Y+**	–	**Y+**	**Y+**	**Y+**
Retinol/Vitamin A (µg)	–	–	–	–	–	–	–	–	–	–	–	–	–	–	–	–	–	–	–	–	–	*Y+*	*Y+*	*Y+*
Carotene (µg)	–	–	–	*Y+*	*Y+*	*Y+*	–	–	–	*Y+*	*Y+*	–	–	–	–	*Y+*	*–*	–	*Y+*	*Y+*	–	**Y+**	**Y+**	**Y+**
Vitamin D (µg)	–	–	–	**Y+**	**Y+**	–	–	*Y+*	–	–	–	–	–	–	–	*Y+*	**Y+**	–	–	–	–	**Y+**	**Y+**	*Y+*
Vitamin E (mg)	–	–	–	–	–	–	*Y*-	–	–	–	*Y+*	–	**Y**-	*Y*-	–	–	–	–	–	*Y+*	–	**Y+**	*Y+*	–
Ca (mg)	–	–	–	–	*Y+*	–	–	–	–	–	–	–	*Y*-	–	–	–	–	–	–	–	–	**Y+**	**Y+**	–
Phosphorus (mg)	–	–	–	*Y+*	**Y+**	–	–	–	–	–	–	–	–	–	–	–	*Y+*	–	–	–	–	**Y+**	**Y+**	*Y+*
Mg (mg)	–	–	–	*Y+*	*Y+*	–	*Y*-	–	–	*Y+*	*Y+*	–	**Y**-	*Y*-	–	–	–	–	*Y+*	**Y+**	–	**Y+**	**Y+**	–
Na (mg)	–	–	–	*Y+*	**Y+**	–	–	–	–	–	–	–	*Y*-	–	–	–	*Y+*	–	–	–	–	**Y+**	**Y+**	*Y+*
Potassium (mg)	–	–	–	*Y+*	**Y+**	*Y+*	–	*Y+*	–	–	–	–	–	–	–	*Y+*	**Y+**	–	–	–	–	**Y+**	**Y+**	*Y+*
Fe (mg)	–	–	–	*Y+*	*Y+*	–	–	–	–	–	*Y+*	*Y+*	–	*Y*-	–	–	–	–	*Y+*	**Y+**	*Y+*	**Y+**	**Y+**	*Y+*
Zn (mg)	–	–	–	**Y+**	**Y+**	–	–	*Y+*	–	–	–	–	–	–	–	*Y+*	**Y+**	–	–	–	–	**Y+**	**Y+**	**Y+**
Se (µg)	–	–	–	–	*Y+*	–	*Y*-	–	–	–	**Y+**	–	**Y**-	–	–	–	–	–	*Y+*	**Y+**	–	**Y+**	**Y+**	*Y+*
Iodine (µg)	–	–	–	**Y+**	**Y+**	–	–	*Y+*	–	–	–	–	–	–	–	*Y+*	**Y+**	–	–	–	–	**Y+**	**Y+**	**Y+**

Results are from adjusted analyses on the imputed data up to number with nutrient data; *n* for mother in pregnancy = 11 812; *n* for mother 4 years post-partum = 9296; *n* for partner at 4 years post-partum = 4786. ‘–’ indicates that the exposure was not associated, ‘Y’ indicates that it was, with + or – denoting the direction of association (i.e. Y + means a positive association; Y- means negative association). Results in bold are based on the Bonferroni-corrected *α* value, results in italics are those with an *α* value less than 0·05 but greater than the Bonferroni-adjusted value. Note that for attendance at a place of worship, results for minimum once a month and minimum once a year have been combined together. Preg = mothers in pregnancy; M4 = mothers 4 years post-partum; P4 = partners 4 years post-partum.

### Mother’s diet 4 years post-partum

#### Dietary patterns

**Fig. 2:**
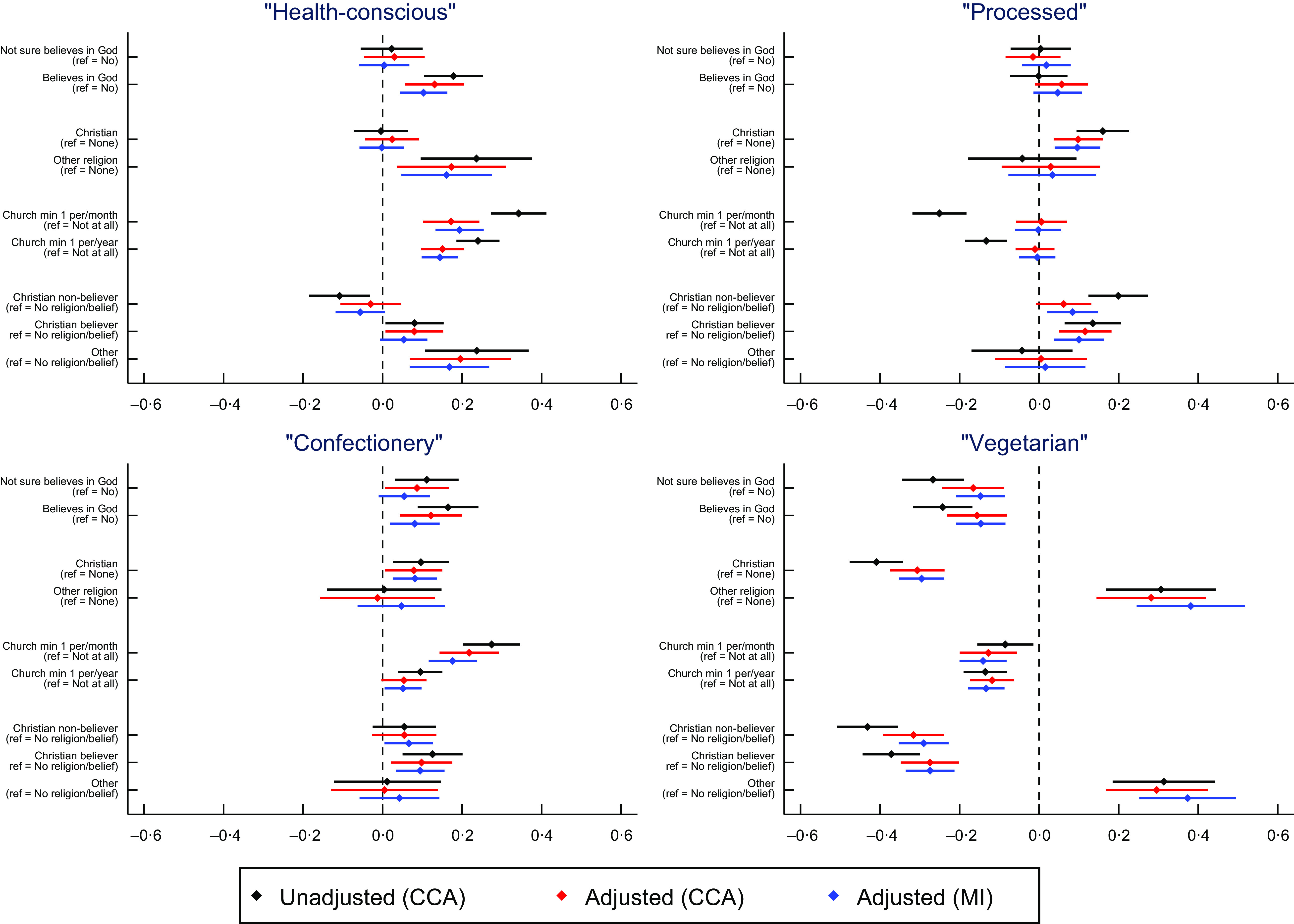
Associations between religious/spiritual beliefs and behaviours and dietary patterns from mothers 4 years post-partum. Positive coefficients indicate a higher score on said dietary pattern. Error bars denote 95 % CI. CCA = complete-case analysis; MI = multiple imputation. *n* for belief in God/divine power CCA = 6312; *n* for religious affiliation CCA = 6239; *n* for attendance at a church/place of worship = 6184; *n* for religious belief and affiliation combined = 6228; *n* for multiple imputation = 13 689

Results for the four dietary pattern scores for the mothers 4 years post-partum are displayed in Fig. 2. As with the pregnancy data, there is little difference in the parameter estimates between the adjusted complete-case models and the imputed data, so we focus on the imputed results. Results for the mother 4 years post-partum are very similar to those during pregnancy, with religious attendance associated with higher ‘health-conscious’ dietary pattern scores, measures of RSBB associated with lower ‘vegetarian’ pattern scores (if Christian) and few strong associations for the ‘processed’ dietary component, although Christian affiliation was somewhat associated with higher ‘processed’ scores. Unlike in pregnancy, all aspects of RSBB associated with higher ‘confectionery’ scores, especially regular religious attendance. Full results are displayed in online supplementary material, Supplemental Table 26.

#### Nutrient intakes

We then explored whether RSBB was associated with specific nutrient intakes, using the adjusted results from the imputed datasets (online supplementary material, Supplemental Figs 6 and 7; Table 3). Results were broadly consistent with the pregnancy findings; RSBB, particularly religious attendance, was associated with a greater intake of micronutrients (Table 4; full results in online supplementary material, Supplemental Tables 27–30). Fewer associations were reported for following recommended nutrient intakes, but again RSBB – and religious attendance in particular – was associated with lower odds of missing several of these recommended daily micronutrient intake guidelines (Table 5; full results in online supplementary material, Supplemental Tables 31–34). When adjusting for ‘total energy intake’, many of these associations were reduced somewhat towards the null, although the majority of associations were still present and the overall patterns of results were similar (online supplementary material, Supplemental Tables 15, 24 and 25; Figs 8 and 9; full results in online supplementary material, Supplemental Tables 35–42).

**Table 5:** Summary of recommended nutrient intake (RNI) results

	Belief in God (ref = no)						Religious affiliation (ref = none)						Belief and religious affiliation (ref = non-religious non-believer)									Attendance at place of worship (ref = not at all)		
	Not sure			Yes			Christian			Other			Christian non-believer			Christian believer			Other			Min once a month/min once a year		
	Preg	M4	P4	Preg	M4	P4	Preg	M4	P4	Preg	M4	P4	Preg	M4	P4	Preg	M4	P4	Preg	M4	P4	Preg	M4	P4
Energy < EAR	–	–	–	–	*Y*-	–	–	–	–	–	–	–	*–*	–	–	–	*Y*-	–	–	–	–	**Y**-	**Y**-	*Y*-
Carbohydrate < min	*Y+*	–	–	–	*Y*-	–	–	*Y*-	–	–	–	–	–	–	–	–	*Y*-	–	–	–	–	*Y*-	**Y**-	*Y*-
Free sugars > max	*Y+*	–	–	–	*Y+*	–	–	*Y+*	–	–	–	–	–	–	–	–	*Y+*	–	–	–	–	–	*Y+*	*Y+*
NSP (fibre) < RNI	–	–	–	–	–	*Y*-	**Y+**	–	–	–	–	–	**Y+**	*Y+*	–	*Y+*	–	–	–	–	*Y*-	*Y*-	*Y*-	*Y*-
Fat > max	–	–	–	–	–	–	–	–	–	–	–	–	–	–	–	–	–	–	–	–	–	*Y+*	*Y+*	–
Monounsaturated fat < RNI	–	–	–	–	*Y*-	–	–	–	–	–	–	–	–	–	–	–	*Y*-	–	–	–	–	*Y*-	*Y*-	–
Polyunsaturated fat < RNI	–	–	–	*Y*-	–	–	–	–	–	–	–	–	–	*Y+*	–	–	–	–	–	*Y*-	–	**Y**-	–	–
Saturated fat > max	–	–	–	–	–	*Y*-	–	–	–	–	–	–	–	–	–	–	–	–	–	–	–	*Y+*	**Y+**	–
*n*-3 from fish < 0·25 g	–	–	–	*Y*-	*Y*-	–	–	–	–	–	–	–	–	–	–	–	*Y*-	–	–	–	–	**Y**-	**Y**-	–
Protein < RNI	–	*Y*-	–	*Y*-	–	–	–	–	–	–	–	–	–	–	–	–	–	–	–	–	–	**Y**-	*Y*-	–
Thiamin < RNI	–	–	–	*Y*-	–	–	–	–	–	–	–	–	–	–	–	–	–	–	–	–	–	**Y**-	–	–
Riboflavin < RNI	*Y*-	–	–	**Y**-	**Y**-	–	–	–	–	–	–	–	–	–	–	–	*Y*-	–	–	–	–	**Y**-	**Y**-	–
Niacin eq. < RNI	–	*Y*-	NA	–	*Y*-	NA	–	–	NA	–	–	NA	–	–	NA	–	–	NA	–	–	NA	–	–	NA
Vitamin B_6_ < RNI	–	–	–	*Y*-	*Y*-	–	–	–	–	–	–	–	–	–	–	–	*Y*-	–	–	–	–	**Y**-	*Y*-	–
Vitamin B_12_ < RNI	–	–	NA	–	–	NA	–	–	NA	*Y+*	–	NA	–	–	NA	–	–	NA	*Y+*	–	NA	–	*Y*-	NA
Folate < RNI	–	–	–	*Y*-	**Y**-	–	–	–	–	*Y*-	–	–	–	–	–	*Y*-	–	–	*Y*-	*Y*-	–	**Y**-	**Y**-	–
Vitamin C < RNI	–	–	–	–	–	*Y*-	–	–	–	–	–	–	–	–	–	–	–	–	–	–	–	**Y**-	*Y*-	–
Retinol/Vitamin A < RNI	–	–	–	–	–	–	–	–	–	–	–	–	–	–	–	–	–	–	–	–	–	**Y**-	*Y*-	*Y*-
Vitamin D < RNI	–	NA	NA	–	NA	NA	–	NA	NA	–	NA	NA	–	NA	NA	–	NA	NA	–	NA	NA	*Y*-	NA	NA
Vitamin E < min	*Y*-	–	NA	*Y*-	–	NA	–	–	NA	–	–	NA	–	–	NA	–	–	NA	–	–	NA	**Y**-	*Y*-	NA
Ca < RNI	–	–	–	–	–	–	–	–	–	–	–	–	*Y+*	–	–	–	–	–	–	–	–	**Y**-	**Y**-	–
Phosphorus < RNI	–	*Y*-	NA	–	–	NA	–	–	NA	–	–	NA	–	–	NA	–	–	NA	–	–	NA	–	–	NA
Mg < RNI	–	–	–	–	–	–	–	–	–	–	*Y*-	–	**Y+**	*Y+*	–	–	–	–	–	*Y*-	–	**Y**-	**Y**-	*Y*-
Na < RNI OR > max	–	–	–	–	*Y+*	–	–	–	–	–	–	–	–	–	–	–	–	–	–	–	–	–	**Y+**	*Y+*
Potassium < RNI	–	–	–	–	**Y**-	–	–	*Y*-	–	–	–	–	–	–	–	–	**Y**-	–	–	–	–	**Y**-	**Y**-	*Y*-
Fe < RNI	–	–	–	–	–	–	–	–	–	–	–	–	–	–	–	–	–	–	*Y*-	–	–	**Y**-	**Y**-	–
Zn < RNI	–	–	–	*Y*-	**Y**-	–	–	–	–	–	–	–	–	–	–	*Y*-	*Y*-	–	–	–	–	**Y**-	**Y**-	*Y*-
Se < RNI	–	*Y*-	–	*Y*-	**Y**-	–	–	–	–	–	–	–	*Y+*	–	–	–	–	–	–	*Y*-	–	**Y**-	**Y**-	–
Iodine < RNI	–	–	–	**Y**-	**Y**-	–	–	–	–	–	–	–	–	–	–	*Y*-	**Y**-	–	–	–	–	**Y**-	**Y**-	**Y**-

Results are from adjusted analyses on the imputed data up to number with nutrient data; *n* for mothers in pregnancy = 11 812; *n* for mothers 4 years post-partum = 9296; *n* for partners 4 years post-partum = 4786. EAR = estimated average requirement; RNI: = reference nutrient intake. ‘–’ indicates that the exposure was not associated, ‘Y’ indicates that it was, with + or – denoting the direction of association (i.e. Y + means a positive association (e.g. more likely to miss RNI); Y- means negative association (e.g. less likely to miss RNI)). Results in bold are based on the Bonferroni-corrected *α* value, results in italics are those with an *α* value less than 0·05 but greater than the Bonferroni-adjusted value. Note that for attendance at a place of worship, results for minimum once a month and minimum once a year have been combined together. UK governmental recommended intake values were available for twenty-nine of the thirty-three nutrients (see online supplementary material, Supplemental Table 1), with a cut-off of <0·25 g/d used for *n*-3 intake (RNI were not available for carotene, cholesterol, starch or total sugars). Due to small sample sizes for recommended nutrient intakes, it was not possible to assess vitamin D for the mothers 4 years post-partum, or niacin, phosphorous, vitamin B_12_, vitamin D or vitamin E for the partners 4 years post-partum; these time/person-points have 28 and 24 recommended intake comparisons, respectively, compared with the 29 for mothers in pregnancy, and are marked with an ‘NA’ in the table below. Preg = mothers in pregnancy; M4 = mothers 4 years post-partum; P4 = partners 4 years post-partum.

### Partner diet 4 years post-partum

#### Dietary patterns

**Fig. 3:**
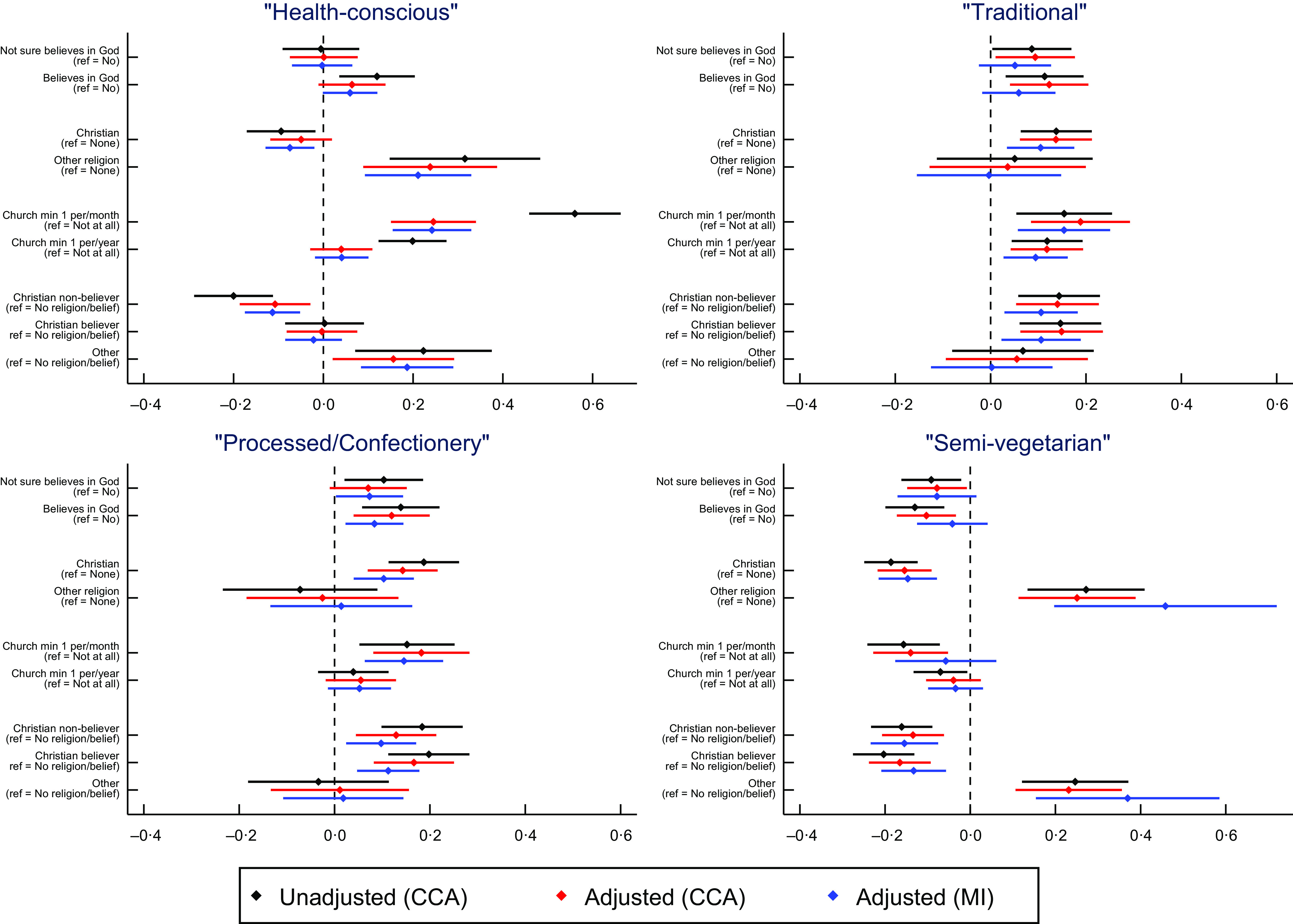
Associations between religious/spiritual beliefs and behaviours and dietary patterns from partners 4 years post-partum. Positive coefficients indicate a higher score on said dietary pattern. Error bars denote 95 % CI. CCA = complete-case analysis; MI = multiple imputation. *n* for belief in God/divine power CCA = 3414; *n* for religious affiliation CCA = 3360; *n* for attendance at a church/place of worship = 3356; *n* for religious belief and affiliation combined = 3355; *n* for multiple imputation = 13 689

Results for the four dietary pattern scores for the partners 4 years post-partum are displayed in Fig. 3. Although some of the dietary components differ slightly, overall the patterns are similar to the mothers’ results (again focusing on results from imputed datasets): attending a place of worship at least once per month was associated with higher ‘health-conscious’ dietary pattern scores; Christians were associated with lower ‘semi-vegetarian’ scores; measures of RSBB were somewhat associated with higher ‘traditional’ scores, while RSBB overall was weakly associated with higher ‘processed/confectionery’ scores (see online supplementary material, Supplemental Table 43 for full results).

#### Nutrient intakes

Next, we explored whether RSBB was associated with specific nutrients and recommended nutrient intakes, using the adjusted results from the imputed datasets (online supplementary material, Supplemental Figs 10 & 11; Table 3). Compared with the mothers’ results, fewer associations were reported for the partners, with only religious attendance associated with increased micronutrient intake at the Bonferroni-corrected *α* level (Table 4; full results in online supplementary material, Supplemental Tables 44–47). Few associations were reported for following recommended daily nutrient intake guidelines, with only one nutrient associated with any RSBB exposure at the Bonferroni-corrected level (religious attendance was associated with lower odds of missing iodine recommended intake; Table 5; full results in online supplementary material, Supplemental Tables 48–51). Similar results were found when adjusting for ‘total energy intake’, although again many effect sizes were attenuated towards the null (online supplementary material, Supplemental Tables 15, 24 and 25; Figs 12 and 13; full results in online supplementary material, Supplemental Tables 52–59). These differences compared with the mother’s results appear to be in part due to both smaller sample sizes for the partner nutrient analyses (4786 *v* 11 812 and 9296 for mothers in pregnancy and 4 years post-partum, respectively) resulting in less precise estimates and smaller effect sizes for partners.

## Discussion

In a large population-based longitudinal UK cohort of mothers and their partners, we found numerous associations between RSBB and diet. Looking at dietary patterns, RSBB was associated with substantially lower ‘vegetarian’ pattern scores (if Christian) and somewhat higher ‘traditional’ (‘meat and two veg’) dietary pattern scores. Results for ‘health-conscious’ dietary pattern scores were more complex, with attendance at a place of worship, belief in God/divine power and non-Christian religious affiliation associated with higher ‘health-conscious’ dietary pattern scores, while Christian religious affiliation was associated with somewhat lower scores. Few associations between RSBB and ‘processed’ or ‘confectionery’ dietary pattern scores were found. These findings were broadly similar for the mothers at both time points (pregnancy and 4 years post-partum) and for the partners (4 years post-partum). Consistent with previous research linking dietary patterns to specific nutrient intakes^(33)^, we observed that RSBB was generally associated with both increased micronutrient intake and being more likely to follow recommended daily micronutrient intake guidelines, but the associations were less clear for partners (see Tables 3, 4 and 5 for summaries). Compared with belief in God/divine power and religious affiliation, religious attendance was a much stronger predictor of nutrient intakes in both mothers and partners. These patterns of nutrient intake results were largely robust when adjusting for ‘total energy intake’, although many effect estimates, especially for attendance at a place of worship, were somewhat attenuated towards the null (i.e. no association).

In these analyses, we attempted to adjust for all hypothesised confounders of the RSBB–diet association, although it is of course possible that other unmeasured variables may confound this relationship. In addition to adjusting for relevant potential confounders, we also performed multiple imputation to explore whether selection bias due to missing data may impact our complete-case analysis results, using a range of auxiliary variables in an attempt to make the missing at random assumption more plausible. We observed little difference between the complete-case analyses and those from the imputed datasets. This suggests that either there is little bias in our complete-case analyses or that both analyses were equally biased and that our choice of auxiliary variables was not sufficient to remove potential biases caused by selection; from the observed data we are unable to distinguish between these alternatives. Nonetheless, these results are consistent with the hypothesis that RSBB may be a causal factor shaping diet, although such conclusions must remain tentative until further research has been conducted; replication of these results, especially in studies with different confounding or selection structures, would bolster these conclusions.

Assuming that these associations are causal, our results suggest that diet has the potential to be a mediator on the pathway between RSBB and health outcomes^(2,3,35)^. Given that many facets of RSBB – other than Christian affiliation – were associated with both higher ‘health-conscious’ dietary pattern scores (this pattern is associated with increased intake of healthy foods such as pulses, fish, salad and wholegrain cereals) and increased intake of micronutrients (including Ca, folate, *n*-3, iodine, Fe, Mg, potassium and Zn), these suggestive results warrant further investigation. Previous longitudinal studies have suggested that both diet and some of these micronutrients may be especially important during pregnancy for child outcomes^(9)^. While these results are suggestive, we stress that they do not demonstrate that these differences in diet and nutrient intake translate into meaningful health differences and that additional research is required to explore these links further. Furthermore, few strong or consistent differences by RSBB were observed for dietary patterns linked to high-fat and/or −sugar intake (such as ‘confectionery’ or ‘processed’ dietary patterns); as these diets have the strongest association with negative health outcomes^(5,6)^, the extent to which the patterns reported here may impact subsequent health is uncertain.

This article has shown that complex social behaviours such as RSBB are associated with differences in dietary patterns and nutrient intakes. Previous research on the associations between RSBB and diet has provided mixed results^(19,21–23)^, although in general religion appeared somewhat associated with potentially healthier diets^(2)^. Our findings bolster this tentative conclusion but suggest that religious attendance, rather than religious beliefs or affiliation, has a stronger association with dietary patterns and nutrient intake. This is consistent with wider evidence that the social elements of religion – such as participation in religious services – have a larger impact on health and behaviours than personal aspects of religion such as religious beliefs or identity^(1)^, although the mechanism(s) by which these social effects work is unclear. Understanding how RSBB, and religious attendance in particular, translate into differences in diet is a key area for future research. For instance, RSBB may reduce the likelihood of risk-taking behaviours^(1)^, including poor diet^(21)^ (although see^(18)^). Alternatively, research has also suggested that RSBB may be linked to eating disorders – although the evidence suggests that RSBB could both protect and exacerbate such symptoms^(53)^ – which may also mediate any relationship between RSBB and diet. Additional research is needed to explore these potential mediators in more detail.

### Strengths and limitations

A key strength of this research is the use of a large population-based cohort with detailed measures of diet assessed at multiple time points. This is an improvement on much previous research in this area, which tended to have small sample sizes, cross-sectional designs and crude measures of diet^(2,19,21–23)^. That similar patterns of results, both for dietary patterns and nutrient intakes, were replicated in the mothers at both time points (and to some extent in the partners as well) also suggests that these results are relatively robust.

A further strength is the detailed baseline data collected in this cohort, allowing for a range of potential confounders of the RSBB–diet relationship to be controlled for. As mentioned above, however, there is the potential for unmeasured confounding and selection bias; we have attempted to account for these as best as possible, but it is always possible in observational research that unmeasured sources of bias remain. For instance, in our choice of confounders we excluded variables hypothesised to be potential mediators of the RSBB–diet relationship, such as smoking, alcohol intake and mental health status. While these are all likely caused by RSBB – and hence mediators – it is possible that some may also cause RSBB (e.g. alcohol intake may lead to lower religious attendance), meaning that they may also be confounders. In these situations of reciprocal causation where covariates are both confounders and mediators, causal inference from cross-sectional designs will not be possible^(4,42)^. Replication in similar longitudinal population studies, and making use of longitudinal data on exposures and covariates, would provide corroborating support.

A potential limitation of this research is that these dietary data were obtained from FFQ. It is possible that participants may not accurately recall how frequently they consumed certain foods, or intentionally under-report certain ‘undesirable’ food items, while the use of average portion sizes to derive nutrient intakes may not reflect actual food intake, which together may contribute to measurement error and potential bias. However, these FFQ measures have performed well against more sensitive diet diary measures, both in terms of nutrient profiles^(28)^ and dietary pattern principal components^(31)^.

A further limitation is that the extent to which these results are generalisable beyond the study population – Bristol-based UK parents in the early 1990s who are predominantly Christian (the majority of which are Protestant) – is unknown. There are large cultural and historical differences in religion and diet, meaning the association between RSBB and diet may vary considerably both cross-culturally and temporally. As discussed in the introduction, compared with many other cultures and religions, Christianity imposes relatively few dietary restrictions on its adherents^(14)^; differences in diet between non-believers and other faiths may therefore be greater, and patterned differently, than observed here. As most previous work in this area was conducted on predominantly Christian Americans in the late 1900s/early 2000s, understanding how culturally and historically variable these results are is a key area for future research.

This study also grouped together many different beliefs. Although ∼80 % of the Christian sample were Protestant/Church of England, it is possible that there are differences between Christian denominations which were not explored here; for instance, although eating fish on a Friday used to be common in the UK, it originated as a Catholic practice, so differences in diet between Christian denominations may have been overlooked. We also lack the detailed information on religious observance to know whether, for example, Christian individuals practised either Lent or ‘Friday fasting’, which may mediate the RSBB–diet relationship. Results from the ‘other’ religious affiliation category used here should also be interpreted with caution, as this is not a well-defined group and contains individuals from many disparate faiths including Judaism, Buddhism, Sikhism, Hinduism and Islam, as well as a majority (∼75 %) who self-identify as ‘other’. These belief systems have very different rules and stipulations regarding diet, and although differences in diet for this ‘other’ category are suggestive (as they are associated with higher ‘health-conscious’ and ‘vegetarian’ dietary pattern scores), additional research using larger samples of these groups is necessary before conclusions can be made regarding these non-Christian faiths/belief systems.

## Conclusion

These results suggest that, in a Bristol-based cohort of parents in the early 1990s, RSBBs – and attendance at church/place of worship in particular – are associated with differences in broad dietary patterns and specific nutrient intakes. Further research is required to assess whether these patterns may be causal and whether these differences in diet translate into meaningful differences in health and hence investigate the role of diet as a mediator between RSBB and subsequent health outcomes.

## Data Availability

ALSPAC data access is through a system of managed open access. Information about access to ALSPAC data is given on the ALSPAC website (http://www.bristol.ac.uk/alspac/researchers/access/). The datasets presented in this article are linked to ALSPAC project number B3893, please quote this project number during your application. Analysis code is openly-available here: https://github.com/djsmith-90/AnalysisCode_ALSPACG0DietPatterns_B3893.

## Supplementary material

For supplementary material accompanying this paper visit https://doi.org/10.1017/S1368980023001866
